# Myricetin: A Significant Emphasis on Its Anticancer Potential via the Modulation of Inflammation and Signal Transduction Pathways

**DOI:** 10.3390/ijms24119665

**Published:** 2023-06-02

**Authors:** Arshad Husain Rahmani, Ahmad Almatroudi, Khaled S. Allemailem, Wanian M. Alwanian, Basmah F. Alharbi, Faris Alrumaihi, Amjad Ali Khan, Saleh A. Almatroodi

**Affiliations:** 1 Department of Medical Laboratories, College of Applied Medical Sciences, Qassim University, Buraydah 51452, Saudi Arabia; 2 Department of Basic Health Sciences, College of Applied Medical Sciences, Qassim University, Buraydah 51452, Saudi Arabia

**Keywords:** myricetin, apoptosis, inflammation, cancer therapy, signal transduction pathways

## Abstract

Cancer is a major public health concern worldwide and main burden of the healthcare system. Regrettably, most of the currently used cancer treatment approaches such as targeted therapy, chemotherapy, radiotherapy and surgery usually cause adverse complications including hair loss, bone density loss, vomiting, anemia and other complications. However, to overcome these limitations, there is an urgent need to search for the alternative anticancer drugs with better efficacy as well as less adverse complications. Based on the scientific evidences, it is proven that naturally occurring antioxidants present in medicinal plants or their bioactive compounds might constitute a good therapeutic approach in diseases management including cancer. In this regard, myricetin, a polyhydroxy flavonol found in a several types of plants and its role in diseases management as anti-oxidant, anti-inflammatory and hepato-protective has been documented. Moreover, its role in cancer prevention has been noticed through modulation of angiogenesis, inflammation, cell cycle arrest and induction of apoptosis. Furthermore, myricetin plays a significant role in cancer prevention through the inhibition of inflammatory markers such as inducible nitric oxide synthase (iNOS) and cyclooxygenase-2 (Cox-2). Moreover, myricetin increases the chemotherapeutic potential of other anticancer drugs through modulation of cell signaling molecules activity. This review elaborates the information of myricetin role in cancer management through modulating of various cell-signaling molecules based on in vivo and in vitro studies. In addition, synergistic effect with currently used anticancer drugs and approaches to improve bioavailability are described. The evidences collected in this review will help different researchers to comprehend the information about its safety aspects, effective dose for different cancers and implication in clinical trials. Moreover, different challenges need to be focused on engineering different nanoformulations of myricetin to overcome the poor bioavailability, loading capacity, targeted delivery and premature release of this compound. Furthermore, some more derivatives of myricetin need to be synthesized to check their anticancer potential.

## 1. Introduction

Cancer is a multifactorial disease and it has emerged as a significant disorder accountable for a large number of deaths yearly worldwide [1]. More than 19.3 million new cancer cases are diagnosed and reported recently, leading to almost 10 million deaths based on the reported data [2]. In spite of the development of innumerable treatment approaches, cancer remains a key cause of death worldwide [3,4]. The current mode treatment for cancer patients is important, but these types of treatments cause some serious adverse effects such as severe nausea and vomiting [5].

In this vista, approximately 50% of accepted cancer therapeutic agents are derived from natural products as well as, secondarily; medicinal plant metabolites have established a valuable perspective as a source of anti-cancer as well as chemo-preventive compounds [6]. Moreover, natural products have gathered growing consideration in cancer chemotherapy as they are regarded as more biologically responsive and therefore more co-evolved with their target sites as well as less toxic to normal cells [7]. Moreover, consumption of natural products or their bioactive compounds is associated with a low risk of various pathogenesis including cancer. Several studies have proven role of natural compound or their bioactive compounds in the inhibition of pathogenesis including cancer through modulation of various biological activities [8,9,10,11].

Various experimental studies exhibited that natural products presented inhibitory potentials for cancer prevention via inhibiting proliferations, angiogenesis, cell migrations, induction of apoptosis, and arresting the cell cycle [12,13,14,15]

**Figure 1:**
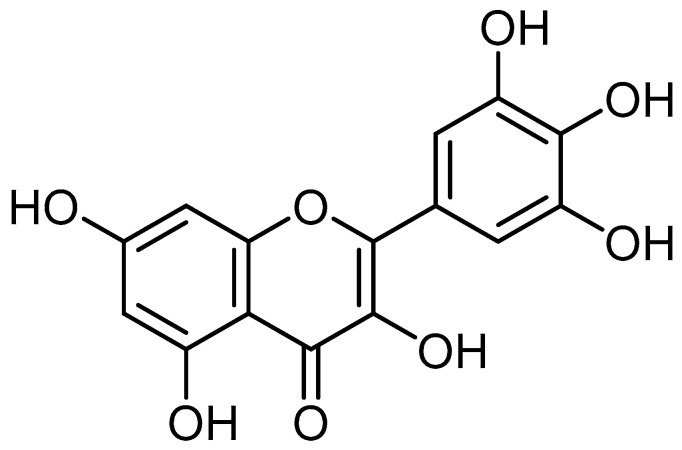
Chemical structure of myricetin.

Myricetin (3, 5, 7, 3′, 4′, 5′-hexahydroxyflavone), an important flavonoid and chiefly found in the glycoside form (*O*-glycosides) in fruits, vegetables, berries, nuts, herbs, plants, beverages and medicinal plants [16,17,18,19,20,21,22] (Figure 1).

Previous finding supports the role of myricetin with antioxidant [23], anti-inflammatory [24] hepato-protective [25] and anti-diabetic potential in diseases management [26]. Moreover, its role in various types of cancer has been explained through modulation of various important cell-signaling molecules [27]. The recent studies demonstrate the potential role of myricetin in cancer management through various hallmarks of cancer through different molecular mechanisms employed by this flavonoid to mitigate cell proliferation, angiogenesis, metastasis, and induction of apoptosis [28,29] and its role in inhibition of other pathogenesis has been described [30].

This review summarizes the evidences of myricetin role in cancer management by modulating different cell-signaling molecules based on in vitro and in vivo studies. In addition, the synergistic effect with some anticancer drugs and approaches to enhance its pharmacokinetics is discussed. The facts compiled in this review will help different health professionals to comprehend the knowledge about its safety aspects, mechanism of action, effective dose against cancers and implication in clinical trials.

### Origin and Quantity of Myricetin in Different Plants

From the plants of Myricaceae, *Comptonia peregrina* (L.) *Coult.* and *Morella cerifera* (L.) Small, first time myricetin was discovered [31,32]. The most common sources of myricetin are fruits, vegetables, berries, nuts, as well as tea [33]. *Rosa canina* L. (rosa hip), *Urtica dioica* L. (nettle), and *Portulaca oleracea* L. (purslane) plants contains myricetin concentration in the range between 3 and 58 mg/kg [20]. Other sources of myricetin are bambara groundnut (*Vigna subterrania*) with 1800 mg/g [34] and in the *Lycium barbarum* L. fruits, 57.2 mg of myricetin/g [35]. Myricetin is also present in different vegetables and fruits as blueberry (25 mg/kg) [19], Crowberry (44mg/kg) [19], green chilli (11.5 mg/kg) [36], red chilli (29.5 mg/kg) [36], garlic (693 mg/kg) [36], guava (549.5 mg/kg) [36]. Other study reported that carrot (525.3 mg/kg), spinach (1660.9 mg/kg), turnip (457.0 mg/kg), cauliflower (1586.9 mg/kg), and peas (146.2 mg/kg) contain myricetin [16].

## 2. Mechanism of Action of Myricetin in Cancer Prevention

**Figure 2:**
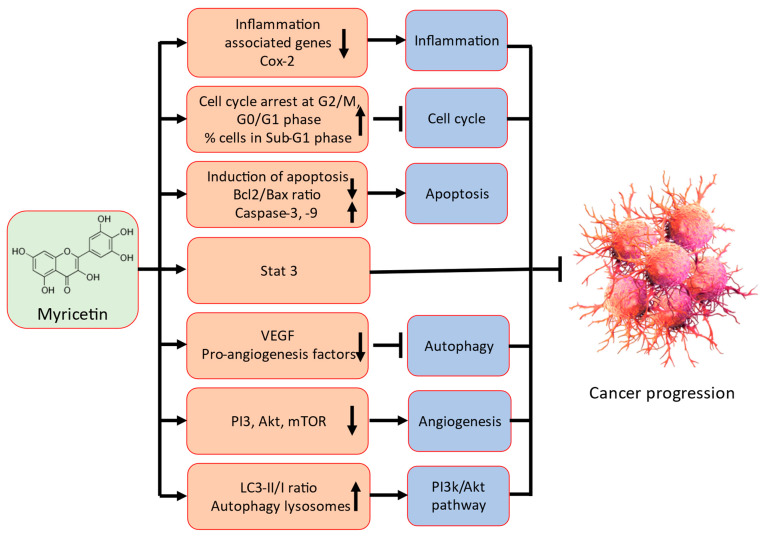
Possible mechanism of action of myricetin in cancer prevention.

Myricetin has been identified as having anti-cancer potential through modulation of a variety of cell signalling molecules and pathways, including inflammation, apoptosis, cell cycle, PI3K/Akt, angiogenesis, transcription factor/components, and also other compound or molecules (Figure 2).

Detailed possible mechanism of action of myricetin in cancer prevention are described in the following sections.

### 2.1. Inflammation

It is widely understood that viral and bacterial infections, chronic inflammation, and up to 25% of human cancers are linked [26]. Besides this, lipopolysaccharide (LPS), is a commonly used compound to induce the inflammation in experimental animals. It is significantly associated with chronic intestinal inflammation and initiates cancer progression. A crosstalk exists between LPS/TLR4 signal transduction pathway and different metabolic pathways including primary bile acid biosynthesis and secretion, renin-angiotensin system, arachidonic acid pathways and glutathione metabolism, important in driving chronic intestinal inflammation and intestinal carcinogenesis [37].

The hallmark in the commencement and proliferation of cancer is also characterized by inflammation associated with cancer [38]. Furthermore, the fact that significant pro-inflammatory transcription factors like nuclear factor-κB (NF-κB) and signal transducers and activators of transcription 3 can be induced via a multitude of these dynamic cancer risk factors clearly illustrates the crucial relationship between chronic inflammation and cancer [39]. The chronic inflammatory microenvironment is predominated by build-up of macrophages. The macrophages, in combination with other leukocytes, produce excessive reactive oxygen species (ROS) and reactive nitrogen species (RNS) to fight infection [40]. However, a continuous tissue damage and cellular proliferation leads to the persistence of these infection-fighting agents, which is deleterious. Mutagenic agents are usually produced during this phenomenon. The different mutagenic agents include peroxynitrite, which cause DNA mutation in proliferating epithelial and stroma cells. Macrophages and T-lymphocytes may release tumor necrosis factor-α (TNF-α) and macrophage migration inhibitory factor to exacerbate DNA damage [41]. These overall events lead to cancer development.

Moreover, the chronic inflammation can lead to cancer in target organs as well as several tissues via multiple pathways [42]. Various natural compounds show anti-inflammatory potential via inhibiting the production of inflammatory mediators or related signalling molecules [43,44,45,46,47,48].

A recent study reported that myricetin significantly inhibited cytokine-initiated migration and invasion of cholangiocarcinoma cells. Moreover, the cytokine-initiated upregulation of metastasis and inflammatory-linked genes, which are downstream genes of STAT3 together with the inducible nitric oxide synthase (iNOS), intercellular adhesion molecule-1 (ICAM-1), cyclo-oxygenase 2 (Cox-2) and matrix metalloproteinase-9 (MMP-9) were meaningfully stopped by the treatment of myricetin [49] (Table 1). Myricetin inhibited the occurrence of colorectal tumorigenesis and decrease the size of colorectal polyps [50]. Besides, myricetin could reduce the amount of colonic inflammation and colorectal tumorigenesis. Additional finding exhibited that myricetin powerfully decrease the levels of the inflammatory factors in the colonic tissues. Myricetin holds the biological activities of the chemoprevention of colonic chronic inflammation, as well as inflammation-driven tumorigenesis [50]. Myricetin played role in the inhibition of NLRP3 inflammasome assembly through promotion of ROS-independent ubiquitination of NLRP3 as well as decrease of ROS-dependent ubiquitination of apoptosis-associated speck-like protein comprising a CARD (ASC), which disturbs the interaction between NLRP3 and ASC and prevents the ASC oligomerization [51]. The effects of myricetin on blood concentrations of PGE2 and interleukin-6 (IL-6) as well as pro-inflammatory cytokines (MCP-1, TNF-α, IL-1β, and IL-6) in adenomatous polyps were investigated. Result revealed that both IL-1β precursor as well as mature IL-1β were strongly prevented in small intestinal and colonic polyps. Furthermore, it demonstrated that myricetin decrease the levels of TNF-α by 89.4% and 91.9%, IL-6 by 76.6% and 89.2% and MCP-1 by 77.2% and 83.3% in small intestinal and colonic polyps, correspondingly [52]. Inflammatory cytokine secretions were meaningfully inhibited by myricetin pretreatment. These finding enrich the full bioactivities of myricetin and demonstrate that myricetin has potential anti-inflammatory property [53].

### 2.2. Apoptosis

Apoptosis is defined as a type of controlled cell death initiaed by a programmed cascade of molecular steps. However, when the physiological process tended to be altered, several pathological transformations occur to develop malignancy [54]. Thus, it was confirmed that controlling and induction of apoptosis are possible ways in the treatment of cancer [55,56]. By causing cancer cells to undergo apoptosis, natural compounds have a documented role in cancer prevention. Furthermore, there were apoptotic bodies in the cell and vesicles in the cell membrane. The percentage of apoptotic cells was 28.5 and 67.4% in the 50 and 100 μmol/L myricetin-exposed groups, respectively. Flow cytometry exhibited similar findings, with the apoptotic rate being positively correlated with the concentration of myricetin (50 and 100 μmol/L). Besides, myricetin (25, 50 and 100 μmol/L) reduced the ratio of Bcl-2/Bax and induced apoptosis in a dose-dependent manner [57] (Table 1). A recent study reported that apoptotic bodies in the gastric cancer cells were exposed with myricetin. The number of apoptotic bodies significantly increased as myricetin concentration increased, primarily by 2.0% in the control group, 6.0% in the myricetin (15 µM) treatment group, and 17.7% in the myricetin (25 µM) treatment group. Moreover, expression of anti-apoptosis protein decreased and pro-apoptosis proteins increased in the myricetin treatment groups when compared to control group [58] (Table 1). The apoptosis in myricetin-treated cells was examined to provide an explanation for the cytotoxicity associated with myricetin in ovarian cancer cells. Ovarian cancer cells receiving myricetin treatment showed signs of apoptosis induction. Besides, myricetin (25 µM) treatment caused the apoptotic signal in ovarian cancer cell to increase by about 2.5 times, while the same treatment caused the apoptotic signal in other ovarian cancer cells (OVCAR3) to increase by about 4-fold when compared to untreated cells. The consequences propose that apoptosis is participated in myricetin-initiated cytotoxicity in certain ovarian cancer cells. Furthermore, pro-apoptotic protein was suggestively increased, and the expression of the anti-apoptotic protein decreased in myricetin-treated ovarian cancer cells [59]. Another important study result based on flow cytometry analysis demonstrated that myricetin (0, 50, 100 µmol/L) led to apoptosis in prostate cancer cells. In addition, western blotting result presented that expression levels of the apoptosis-associated proteins cleaved caspase-3 and cleaved caspase-9 were both enhanced in DU145 and PC3 cells after treatment of myricetin (0, 50, 100 µmol/L) [60] (Table 1).

### 2.3. Cell Cycle

Regulation of cell cycle is an important step in cancer prevention and treatment. Natural compounds have proven role in cancer prevention through cell cycle arrest. The question of whether arrest of cell cycle in cancer cells is related to myricetin treatment was explored in an investigation based on hepatocellular carcinoma (HCC). Liver cancer cell (HepG2 as well as Hep3B) treated with myricetin (0, 25, 50 µM) Showed a clear reduction in cell number in the G0/G1 stage, while the cell cycle was observed to be arrested at the G2/M stage. The proliferative nuclei were intensely declined in a concentration-dependent way when cells were treated in myricetin. These outcomes designated that myricetin suppressed cancer cell proliferation, most probably by stopping cell cycle at the G2/M phase [61] (Table 1).

Anticancer potential of the myricetin in A549 lung cancer cells was examined. Among numerous doses of myricetin, 73 μg/mL was more effective to inhibit the cancer cell growth. It also indorsed sub-G1 phase aggregation of cells as well as an equivalent reduction in the fraction of cells entering the S and subsequent phase which designates apoptotic cell death. Myricetin generated huge free radicals and, changed the potential of mitochondrial membrane in A549 cells. These results advised that myricetin shows cytotoxic potential via arresting the progression of cell cycle and ROS-dependent mitochondria-mediated mortality in cancer A549 lung cancer cells [62] (Table 1).

When thyroid cancer cells were in the sub-G1 stage, myricetin caused a concentration-dependent arrest of growth. In comparison to untreated control cells, the proportion of cells in the sub-G1 stage was larger in cells subjected to myricetin. In contrast, cells exposed to myricetin (100 µM) had a percentage of S phase cells of 17.70 ± 7.66% and untreated cells had a percentage of 10.42 ± 2.95%. According to this finding, myricetin’s ability to stop cells in the sub-G1 phase is one of the ways it killed cancer cells [63]. Flow cytometry-based finding revealed that myricetin caused cell cycle arrest and apoptosis in gastric cancer cells. Flow cytometry based study was performed to evaluate the effect of myricetin on the cell cycle in gastric cancer cells. As compared with control groups, the 20 and 40 μmol/L groups showed a lower percentage of cells in S-phase and a higher percentage in G2/M-phase. Compared with the 20 mol/L group, the 40 µmol/L group had a lower percentage of S-phase cells and a higher percentage of G2/M-phase cells. Compared with the control groups, cyclin B1, cyclin D1, CDK1, and CDC25C were meaningfully decreased in the 20 and 40 mol/L groups, with a higher decrease in the 40 mol/L group [64].

### 2.4. Angiogenesis

Angiogenesis shows a vital role in the development and progression in various pathogenesis [65,66,67]. Natural products inhibit cancer development and progression through its anti-angiogenic activity. The potential role of myricetin on ultraviolet B (UVB)-caused vascular endothelial growth factor (VEGF) expression in SKH-1 hairless mouse skin was explored. Result reported that myricetin treatment significantly inhibited UVB-induced expression of VEGF [68]. Inhibition of VEGF levels by myricetin (5 and 20 µM) therapy resulted in VEGF levels of 74.4 and 51.9% in A2780/CP70 cells and 74.9 and 46.9% in OVCAR-3 cells. It was investigated in vitro whether the culture medium of ovarian cancer cells exposed to various concentrations of galangin and myricetin caused tube formation through HUVEC cells. The findings showed that HUVEC cells formed a well-solidified network in vitro and in vitro angiogenesis was promoted by culture media that was conditioned by these cancer cells. After galangin and myricetin treatment, HUVEC networks were shortened significantly. Moreover, galangin and myricetin treatment showed substantial decrease in tube length. VEGF levels were 74.4 and 51.9% in A2780/CP70 cell and 74.9 and 46.9% in OVCAR-3 cell inhibited by 5 and 20 μM myricetin treatment [69] (Table 1). Another choriocarcinoma study found that myricetin intervention lowered the pro-angiogenic and invasive activity of malignant JAR as well as JEG-3 trophoblast cells via the phosphatidyl inositol-3-kinase (PI3K)/AKT and mitogen activating protein kinase (MAPK) signalling cascades. Further, cell invasion was suppressed approximately 90% by 20 mM myricetin in both JAR and JEG-3 cells. In conditioned medium from JAR cells, the concentration of VEGFA was decrease 40% in response to myricetin [70].

### 2.5. PI3K/Akt Pathways

Alteration of the PI3K/AKT/mTOR signaling pathway by either the mutation or amplification of genes participated in the PI3K pathway, overactivation of RTKs or loss of the tumor suppressor PTEN, has been noticed in several cancer cells, causal to tumor progression as well as metastasis [71,72,73,74]. Natural substances have demonstrated a role in the prevention of cancer by inhibiting the PI3K/AKT signaling cascade. Myricetin therapy demonstrated a contribution to decreasing colon cancer cell line proliferation. Additionally, myricetin (50 and 100 μmol/L) induced cell apoptosis and autophagy by inhibiting PI3K/Akt/mTOR signalling pathway [57] (Table 1). Furthermore, myricetin inhibits the PI3K/Akt/mTOR pathway, commencing autophagy and apoptosis, which lowers the survival rate of gastric cancer cells. The expression of p-PI3K, p-Akt and p-mTOR decreased in a concentration-dependent manner in the myricetin (15 and 25 μM) treatment groups as compared to that in the control group. As a result, myricetin appears to inhibit the PI3K/Akt/mTOR pathway in gastric cancer cells, which prevents the growth of cancer cells and promotes cell-protective autophagy as well as in vivo and in vitro apoptosis [44]. Myricetin reduced the levels of phosphorylated p-Akt, p-MAPK, and p-P38 [75] and myricetin induces apoptosis through ROS induction and inhibits cell migration, tube formation, and PI3K/Akt/mTOR signaling in human umbilical vascular endothelial cells. Myricetin (0.25, 0.5, 1.0 µM) attenuated the phosphorylation of both PI3K and PDK1 in a concentration-dependent manner [76]. Another study based on triple-negative breast cancer cells reported that myricetin modulated pro-angiogenic, cell cycle, and invasion effects through the PI3K/Protein kinase B (PKB/also known as AKT) and MAPK signaling pathways [77]. 

### 2.6. Signal Transducer and Activator of Transcription 3 (STAT3)

The role of myricetin on inflammatory cytokine-initiated STAT3 pathway activation of cholangiocarcinoma cells was examined. The results demonstrated that the cytokine mixture such as IL-6 + IFN-γ plus TNF-α caused STAT3 pathway initiation, clearly by an enhancement phosphorylation of STAT3. Exposure of cancer cells with myricetin showed role in the suppression of STAT3 phosphorylation in a dose-dependent way [49] (Table 1). A study using hepatocellular carcinoma (HCC) cells was carried out to determine if what myricetin causes autophagy and cell cycle arrest in HCC cells by modulating the MARCH1-regulated p38 MAPK/Stat3 signalling pathway. Myricetin (0.25, 0.5, 1.0 µM) treatment has been shown to restrict the expression of p-Stat3 and p-p38 MAPK in cancer cells [61].

### 2.7. Autophagy

Autophagy has dual effect on cancers, which may protect cancer cells from extreme nutrient conditions, whereas it also causes destruction of energy homeostasis as well as kill cancer cells [78]. Natural compounds or their bioactive ingredient play role in the modulation of autophagy. Vacuoles were seen in the myricetin-exposed gastric cancer cells, in the myricetin treatment groups showed higher number of vacuoles. Furthermore, autolysosomes were recognized in the gastric cancer cells. With an increment in myricetin concentration, the amount of positively stained cells gradually enhanced, while in the control group, very few positively stained cells were seen.

The myricetin treatment groups displayed an important increase of the ratio of LC3-II/LC3-I as well as concentrations of protein named as beclin 1 when compared to control group [58]. Whether myricetin caused autopaghy in liver cancer HCC cells was examined. Western blotting was used to analyse the levels of p62, LC3-I, and LC3-II in SMMC-7721 and Hep3B cells. It was observed that there was a significant improvement in the ratio of LC3-II/LC3-I and a dose-dependent decrease in the protein level of p62. To further verify this finding, Human Hepatocellular carcinoma (HCC) cell lines SMMC-7721 and Hep3B cells had been transfected with GFP-LC3 plasmids and were strongly expressing GFP-LC3 were exposed to myricetin at various concentrations (0, 100, and 200 μM). As a result, it was observed that there was an intentional increase in the percentage of cells forming GFP-LC3 puncta in a dose-dependent manner (0, 100, and 200 μM) [79] (Table 1). An important study was performed to evaluate whether myricetin caused autophagy in HepG2 cells. After treatment with myricetin (0, 20, 50 μM) for 24 h, GFP-LC3 changed from the dispersion state to a punctuate state, indicating that myricetin can convert the soluble form of LC3-I to the lipidated and autophagosome-associated form LC3-II, therefore promoting autophagy. Moreover, myricetin treatment enhanced the LC3-II level in a dose-dependent way. In the meantime, the expression of essential autophagic protein PI3K III was increased as well, along with the reduced expression of anti-apoptotic protein [80].

**Table 1:** Mechanism of action of myricetin in cancer prevention.

Pathways	Cell Line	Cancer	Mechanism/Outcomes	Refs.
Inflammation	KKU-100	Cholangiocarcinoma	Myricetin suggestively inhibited cytokine-initiated migration and invasion of cancer cells. The cytokine-initiated upregulation of metastasis and inflammatory-linked genes were meaningfully stopped by the treatment of myricetin	[49]
Apoptosis	HCT116 and SW620	Colorectal cancer	Myricetin decreased the Bcl-2/Bax ratio and caused apoptosis in a concentration-dependent manner. Cancer cells exposed with myricetin showed apoptotic cells became rounder and smaller	[57]
	AGS	Gastric cancer	As compared to 28.93% in the control group, the percentage of apoptotic cells were significantly increased to 39.94% in the group treated with myricetin.	[58]
	A2780 and OVCAR3	Ovarian cancer	Myricetin treatment was seen to induce apoptosis in cancer cells. Pro-apoptotic protein was suggestively increased, and the expression of the anti-apoptotic protein decreased in myricetin-treated group	[59]
	DU145 and PC3	Prostate Cancer	Myricetin pointedly brought apoptosis. Constantly, expression levels of the apoptosis-associated proteins cleaved caspase-3 as well as cleaved caspase-9 after treatment of myricetin	[60]
Cell cycle	Hep3B and HepG2	Hepatocellular cell carcinoma	Cell cycle arrest in HCC cells due to myricetin occurred at the G2/M stage. Myricetin inhibited the proliferation of cancer cells, presumably through blocking the cell cycle just at G2/M stage.	[61]
	A549	Lung cancer	Myricetin induced sub-G1 phase aggregation of cells and reduce in the fraction of cells incoming the S as well as subsequent phase	[62]
	SNU-80 and HATC cell	Human Anaplastic Thyroid Cancer	The proportion of cells present in the sub-G1 stage and S stage was higher in those cells that were exposed with myricetin as compared to untreated control cells	[63]
	GC HGC-27 and SGC7901 cells	Gastric cancer	Myricetin influenced apoptosis as well as cell cycle arrest of gastric cancer cells via regulating related proteins	[64]
Angiogenesis	A2780/CP70 and OVCAR-3 cells	Ovarian cancer	Vascular endothelial growth factor (VEGF), a mediator of angiogenesis, was inhibited by myricetin and galangin in cancer cells.	[69]
	JAR and JEG-3	Choriocarcinoma	Pro-angiogenic and invasive activity of malignant JAR as well as JEG-3 trophoblast cells were reduced by treatment of myricetin	[70]
PI3K/Akt	HT-29, HCT116, SW480 and SW620	Colorectal cancer	Myricetin caused cell apoptosis as well as autophagy through preventing PI3K/Akt/mTOR signalling pathway	[57]
	AGS	Gastric cancer	Myricetin inhibits the PI3K/Akt/mTOR pathway, which lowers the rate of survival of gastric cancer cells.	[76]
STAT3	KKU-100	Cholangiocarcinoma	Exposure of cancer cells with myricetin showed role in the suppression of STAT3 phosphorylation in a dose-dependent way	[49]
Autophagy	Hep3B and HepG2	Hepatocellular carcinoma	Intervention with myricetin decreases the expression of p-Stat3 and p-p38 MAPK in cancer cells.	[61]
	AGS	Gastric cancer	Vacuoles were seen in the myricetin-exposed gastric cancer cells, in the myricetin treatment groups showed higher number of vacuoles.	[58]
	SMMC-7721 and Hep3B cells	Hepatocellular carcinoma	LC3-II/LC3-I ratio was significantly increased. However, the protein level of p62 was clearly lowered in a concentration-dependent way.	[79]

## 3. Myricetin: Potential Role in Different Types of Cancer

**Figure 3:**
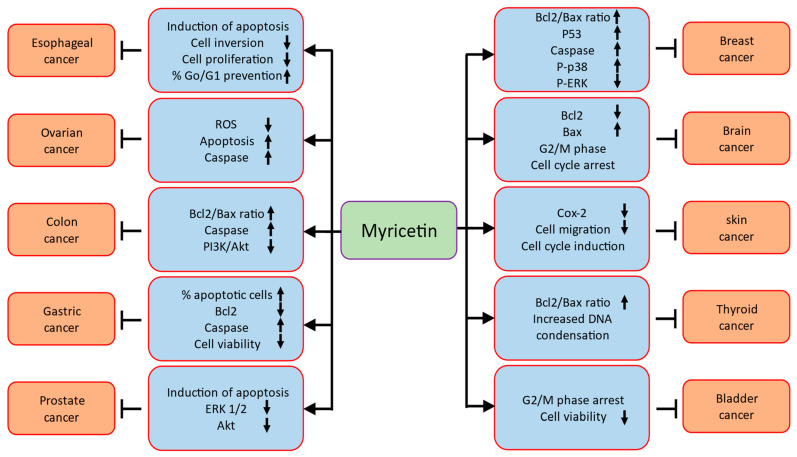
Role of myricetin in different of types of cancers through modulation of cell signalling molecules.

Various experimental studies exhibited that natural products presented inhibitory potentials for cancer prevention via inhibiting proliferations, angiogenesis, cell migrations, induction of apoptosis, and arresting the cell cycle [12,13,14,15] (Figure 3).

Role of myricetin in different types of cancers are described as (Table 2).

### 3.1. Prostate Cancer

Prostate cancer is the second most frequent malignancy (after lung cancer) in men worldwide, counting 1,276,106 new cases and causing 358,989 deaths [81]. Myricetin has established its role in prostate cancer growth inhibition through modulation of various cell-signalling pathways. Flow cytometry analysis exhibited that myricetin meaningfully induced apoptosis in prostate cancer cells. In the meantime, lower apoptosis rates were noticed in RWPE-1 cells, in accordance with the cytotoxicity assays. Consistently, Western Blotting analysis exhibited that the expression levels of the apoptosis-related proteins cleaved caspase-3 and cleaved caspase-9 were both upregulated in PC3 and DU145 cells after myricetin treatment. Also, myricetin inhibited the phosphorylation of ERK1/2 and AKT in PC3 and DU145 cells [60]. A recent and important study reported that myricetin is a potent α-ketoglutarate-type inhibitor that significantly reduces the proliferation of both androgen-dependent and androgen-independent CRPC by inhibiting demethylation activity via KDM4s (C4-2B and CWR22Rv1). Enzalutamide and myricetin have been found to have a synergistic cytotoxic effect on C4-2B. Additionally, in the C4-2B xenograft model, PLGA-myricetin, enzalutamide, and the combination treatment showed significantly better antitumor potential than the control group. Additionally, the co-administration appeared to slow tumor growth compared to enzalutamide or myricetin treatment alone [82].

### 3.2. Colon Cancer

The third most frequent type of cancer is colorectal cancer (CRC) and it is the second foremost cause of cancer death globally [83,84]. Myricetin has proven role in CRC management through modulation of various cell-signalling pathways. Death of human colon cancer cells (HCT-15) was induced after the treatment of myricetin in a dose-dependent means. When compared to controls, 70% decrease in cell viability on human colon cancer cells was noted with the treatment of myricetin (100 μM). To examine apoptotic cell death and DNA damage of colon cancer cells, different concentration of myricetin was used to treat cancer cells. Myricetin (100 μM) treatment of HCT-15 induced substantial nuclear rounding as well as shrinkage of human colon cancer cells as compared to controls. Moreover, effect of myricetin on apoptosis was observed as myricetin enhanced the ratio of BAX/BCL2 as well as BAK expression. However, the expression of procaspase-3 as well as caspase-9 were hardly changed in human colon cancer (HCT-15) cells following myricetin (100 μM) treatment [85]. The anti-cancer effects induced by myricetin treatment in colon cancer HCT-15 cells was evaluated. Based on overall findings, it was proposed that myricetin causes induction of apoptosis in human colon cancer cells selectively via enhancing the expression of NDPK as well as other caspase-regulated apoptosis proteins [86]. The toxic role of myricetin loaded in solid lipid nanoparticles (MCN-SLNs) on the human CRC cells (HT-29) were examined. Result revealed that myricetin loaded in solid lipid nanoparticles could reduce colony numbers as well as survival of the HT-29 cells. The apoptosis index of myricetin loaded in solid lipid nanoparticles-treated cells meaningfully enhanced as compared to the free myricetin. The expression of AIF and Bax were elevated whereas Bcl-2 expression was reduced in myricetin loaded in solid lipid nanoparticles treatment [87]. Another study result revealed that proliferation of four types of colon cancer cell lines was inhibited by myricetin. Moreover, PI3K/Akt/mTOR signalling pathway was inhibited and induction of apoptosis and autophagy was observed after myricetin treatment. Moreover, colon cancer cells exposed to myricetin were induced to undergo apoptosis by the inhibition of autophagy with 3-methyladenine [57].

### 3.3. Liver Cancer

Hepatocellular carcinoma (HCC), the leading variety of liver cancers, accounts the sixth predominant and fourth leading cause of cancer death worldwide [81]. Natural compound including myricetin has recognized role in liver cancer prevention and treatment via modulation of various cell-signalling molecules. To explore the mechanisms underlying myricetin preventing cell growth, proliferation as well as apoptosis of HCC cells treated with myricetin was compared. Result confirmed that proliferation of Huh-7 and HepG2 cells was inhibited by myricetin treatment. Additionally, HepG2 cells treated with myricetin showed significant increased apoptosis rate as compared to control cells and similar pattern was noticed for Huh-7 cells. Besides, myricetin induced Huh-7 and HepG2 cell apoptosis in a time-dependent way. In myricetin-treated HCC cells, cleaved caspase3 levels meaningly increased. Furthermore, other parameters were also checked as myricetin inhibited YAP expression via encouraging its phosphorylation as well as subsequent degradation. Myricetin prevented YAP expression by stimulating LATS1/2kinase activation [88]. To examine the cytotoxic effect of myricetin, HCC cell lines, Hep3B and SMMC-7721 of human HCC cells was used and treated with different concentrations of myricetin. The results demonstrated that myricetin considerably inhibited the proliferation of HCC cells in a concentration- and time-dependent manner. The cytotoxic activity of myricetin was investigated to determine whether it is cancer-selective. The cell line HL-7702, a human normal hepatocyte cell line, was treated with myricetin, and the IC50 values were 252.2 μM (24 h) and 163.9 μM (48 h), which were significantly higher than those of SMMC-7721 and Hep3B cells. Additionally, it appeared that myricetin’s inhibitory activity on HL-7702 cells was relatively weak compared to that of SMMC-7721 as well as Hep3B cells [79].

The role of myricetin on the migration as well as invasion of HCC MHCC97H cells was investigated. It was designated that myricetin reduced the MHCC97H cells viability in a concentration and time dependent way, and migration and invasion of MHCC97H cells was inhibited. As the dose of myricetin increased, lamellipodia and filopodia in cells weakened as well as cells were arranged more nearly. Moreover, myricetin decreased N-cadherin and enhanced E-cadherin expression. Together, the results of the current study establish that myricetin may inhibit the HCC MHCC97H cells migration and invasion by inhibiting the EMT process [89].

Other findings demonstrated that myricetin treatment clearly reduced the viability of HCC cells in a dose-dependent manner. Different doses of myricetin were applied to HCC cells to test whether MARCH1 is involved in the anti-HCC effect. Both Hep3B and HepG2 cells showed a reduction in MARCH1 expression. Additionally, myricetin treatment significantly decreased the number of living HCC cells, especially at the 50 µM concentration. Results also showed that MARCH1 overexpression partially prevented MARCH1 downregulation brought on by myricetin and partially offset the antitumor effect of myricetin. The mRNA level of MARCH1 was interestingly increased by myricetin in Hep3B cells, but significantly decreased in HepG2 cells [61].

### 3.4. Gastric Cancer

The MTT assay was performed to investigate the effect of myricetin on the cell viability of AGS gastric cancer cells. The viability of cancer cells treated with different concentrations of myricetin was 95.8% for 5 µM, 90.3% for 10 µM, 80.6% for 15 µM, and 64.6%, 52.7%, and 36.3% for 20 µM, 25 µM, and 30 µM, respectively. This showed that as the doses of myricetin increased, the viability of gastric cancer cells gradually decreased in comparison to the control group. Apoptosis and autophagy were induced as a result of myricetin inhibition of the PI3K/Akt/mTOR pathway, which also reduced the survival rate of gastric cancer cells. In vivo, a related study was conducted and tumour growth was suppressed [58]. Another study based on flow cytometry analysis exhibited that myricetin induces apoptosis and cell cycle arrest in gastric cancer cells. Moreover, western blotting designated that myricetin influenced apoptosis and cell cycle arrest of gastric cancer cells by regulating associated proteins. SPR analysis presented strong binding affinity of ribosomal S6 kinase 2 (RSK2) and myricetin. Myricetin bound to RSK2, leading to increased expression of Mad1, and contributed to inhibition of HGC-27 and SGC7901 cell proliferation [64].

### 3.5. Pancreatic Cancer

The potential impact of myricetin on pancreatic cancer cells’ viability was assessed, and it was observed that treatment with myricetin significantly decrease cell viability in used pancreatic cancer cells (Panc-1, MIA PaCa-2, and S2-013) in a dose-dependent way, whereas on normal pancreatic ductal cells, mild effect was seen. Additionally, the role of caspase-3 and caspase-9 in myricetin-induced cell death was investigated. Myricetin-containing culture medium was used to treat S2-013 and MIA PaCa-2 for 6 h, which resulted in a significant increase in caspase-3 and -9 activity compared to the control. Additionally, a dose-dependent enhancement of Annexin V positive cells was produced after all tested pancreatic cancer cells were exposed to myricetin for 24 h. This finding supports the notion that apoptosis facilitates myricetin-induced cell death in pancreatic cancer cells. To examine the effects of myricetin on pancreatic cancer growth and local regional spread/metastasis in vivo, it was tested the efficacy of this compound on an orthotopic model of pancreatic cancer. In vivo, treatment of orthotopic pancreatic tumors with myricetin showed tumor regression and decreased metastatic spread [90].

### 3.6. Bile Duct Cancer

Biliary tract cancer is a diverse group of extremely aggressive cancers together with perihilar/intrahepatic/distal cholangiocarcinoma, gallbladder cancer, as well as ampullary cancer [91]. Natural compound including myricetin has documented role in bile duct cancer prevention and treatment via modulation of various cell signalling molecules. A recent study based on cholangiocarcinoma was performed and finding revealed migratory capability initiated by cytokine of cholangiocarcinoma cells exposed with myricetin was evidently stopped in a dose-dependent way [49]. Similarly, myricetin could meaningfully suppress cytokine-induced invasion of cholangiocarcinoma cell. Furthermore, cytokine treatment remarkably enhanced the expression of ICAM-1and MMP-9. Treatment with myricetin meaningfully suppressed cytokine-mediated enhancement of these two genes. Moreover, myricetin also abolished cytokine-caused expression of COX-2 and iNOS, which are the critical molecules, participated in inflammatory as well as carcinogenesis processes [49].

### 3.7. Esophageal Cancer

Myricetin’s potential impact on the chemosensitivity of tumor cells was investigated. It was found that treating esophageal cancer (EC9706) cells with different concentrations of 5-fluorouracil (5-FU) on its own could have a preventive effect on clonogenic survival. However, the surviving fraction significantly dropped when mixed with various myricetin doses. The phases distribution of cell cycle indicated that both 5-FU and myricetin could increase the percentage of EC9706 cells in G0/G1 phase and prevent cells entering into the S phase. Additionally, myricetin or 5-FU treatment of esophageal cancer (EC9706) cells resulted in a notable rise in the G0/G1 stage, which was accompanied by a drop in the S stage. In contrast to myricetin alone (62.1%) or 5-FU alone (68.8%), the percentage of cells in the G0/G1 phase increased significantly when combined with 5-FU to 85.9%. These findings suggest that myricetin may increase 5-FU chemosensitivity on cell cycle arrest in the G0/G1 phase, retard the start of the cell cycle, and inhibit EC9706 cells proliferation. An esophageal cancer EC9706 cell xenograft mice model was used to determine chemosensitizing effect of myricetin in vivo. It was found that myricetin-alone group showed less inhibitory effect on tumor growth, compared with tumors treated with 5-FU only. Though, a significant slower down in tumor growth occurred for mice treated with 5-FU combination with myricetin [92]. Proliferation, apoptosis, and invasion of the esophageal carcinoma cell lines (EC9706 and KYSE30) were examined in relation to the effects of myricetin. Myricetin stopped proliferation as well as invasion and caused the induction of apoptosis of the esophageal carcinoma cell lines. Additionally, it was discovered that myricetin binds RSK2 through the NH2-terminal kinase domain. Further, myricetin demonstrated induction of cell apoptosis through Bad and it was found to suppress the proliferation of KYSE30 and EC9706 cells through Mad1. Through RSK2, myricetin inhibits KYSE30 and EC9706 cells’ proliferative and invasive potential and triggers apoptosis. These findings provide novel insight into the potential of myricetin as a preventive and therapeutic agent for esophageal carcinoma [93].

### 3.8. Ovarian Cancer

Ovarian cancer is one of the utmost common causes of death of cancer among women worldwide and the second most common reason of death from gynecological cancers [81]. CCK-8 cell viability/cytotoxicity assays showed that the optimal concentration range for Myricetin inhibition of SKOV3 proliferation was 1 × 10^−5^–1 × 10^−4^ M for 24 h. At these concentrations, myricetin showed inhibitory role on SKOV-3 cells in a dose-dependent means while not toxic to IOSE-80, non-tumor cells. SKOV3 cells treated with myricetin looked smaller, with a reduced cell, as well as high-rate cell death. Furthermore, ROS levels in these cancer cells were meaningfully decreased by the treatment of myricetin at 10, 20 and 40 μM in a dose dependent way and intracellular MDA was reduced whereas SOD levels enhanced. Comparative to controls, exposure with myricetin meaningfully decreased the number of SKOV3 cells migrating downward as well as invading matrigel in a dose-dependent means [94]. The involvement of apoptosis in myricetin-exposed cells was investigated in order to clarify the basic mechanisms by which myricetin causes cytotoxicity in ovarian cancer cells. It was observed that myricetin treatment promoted apoptosis in OVCAR3 and A2780 cells. Compared to untreated cells, in A2780 cells treated with 25 µM myricetin cells showed an approximately 2.5-fold increase in the apoptotic signal, whereas OVCAR3 cells showed an approximately 4-fold increase. The outcomes advocate that apoptosis is participated in myricetin-caused cytotoxicity in ovarian cancer cells. Additionally, it was also demonstrated that the expression of the anti-apoptotic protein Bcl-2 was decreased and, the expression of a pro-apoptotic protein called as BAX (B-cell lymphoma-2 -associated X-protein) was noticeably elevated in myricetin-treated ovarian cancer cells in the comparison of untreated cells [59]. Viability of SKOV3 cells was inhibited by the administration of myricetin in a concentration-dependent means. Myricetin enhanced the protein levels of active caspase 3 and induced nuclear chromatin condensation and fragmentation. Furthermore, myricetin upregulated ER stress-linked proteins, C/EBP homologous protein and glucose-regulated protein-78 in SKOV3 cells. Phosphorylation of H2AX, a marker of DNA double-strand breaks (DSBs) was found to be enhanced in cells administrated with myricetin. The data designated that myricetin induces DNA DSBs and ER stress, which leads to apoptosis in ovarian cancer SKOV3 cells [95].

### 3.9. Breast Cancer

The apoptotic role of myricetin was investigated on breast cancer cells (MCF-7) to evaluate its possible mechanisms of action. The BAX /Bcl-2 ratio as well as the expression of BRCA, p53, and GADD45 genes and expression levels of apoptosis-associated genes caspase-3, caspase-8, and caspase-9 were meaningfully enhanced following the exposure of breast cancer cells with myricetin. Myricetin efficiently brings apoptosis in breast cancer cells via inducing both intrinsic and extrinsic apoptotic pathways. Myricetin might causes its apoptotic effects on breast cancer (MCF-7) cells through encouraging the BRCA1- GADD45 pathway [96]. Another study indicated that myricetin stimulated the production of hydrogen peroxide (H_2_O_2_) in culture medium devoid of cells as well as in the presence of normal cells and triple-negative breast cancer cells. Furthermore, deferiprone-mediated inhibition of intracellular ROS generation via the iron-dependent Fenton reaction and inhibition of extracellular reactive oxygen species (ROS) accumulation with superoxide dismutase plus catalase inhibited myricetin-induced cytotoxicity in triple-negative breast cancer cell cultures. It was concluded that the cytotoxic effect of myricetin on triple-negative breast cancer cells was through oxidative stress initiated via extracellular H_2_O_2_ produced by autoxidation of myricetin [97].

Myricetin showed an important role in the enhancement of the antioxidant levels in plasma, breast tissue and erythrocyte lysate, and was powerful in inhibiting the oxidative damage caused by the 12-dimethylbenzanthracene (DMBA). Myricetin (50, 100, and 200 mg/kg/oral) treated animal caused comparable outcomes to that of standard vincristine as well as control groups. Myricetin was found to be either equieffective or more powerful than vincristine in all studied parameters [98]. The cancer preventive role of myricetin were established in SK-BR-3 cells (human breast cancer cells). The viability of the cells decreased as myricetin concentration was increased, and apoptosis and apoptotic bodies significantly increased. Furthermore, levels of Bcl-2 were decreased and cleaved PARP and Bax proteins increased. While phosphorylated extracellular regulated kinase (pERK) expression levels were down, they were up for phosphorylated mitogen activated protein kinases (p-p38) and c Jun N terminal kinase (JNK). Besides that, the relationship between cell viability and autophagy in cells administered with myricetin was investigated using 3 methyladenine (3 MA). The findings demonstrated that breast cancer cells were encouraged to undergo apoptosis when given methyladenine and myricetin simultaneously. A JNK inhibitor treatment also reduced cell viability, promoted the expression of Bax, and reduced the expression of p JNK, Bcl 2, and LC 3 II/I [99].

### 3.10. Cervix Cancer

Investigations were conducted into the anticancer potential of myricetin, methyl eugenol, and cisplatin (CP), both individually and together, against cervical cancer cells. The findings showed that, in contrast to single drug therapy, the combined effect of methyl eugenol or myricetin with CP induced a stronger impact through provoking apoptosis and preventing growth of cancer cell. The combination of myricetin or methyl eugenol with CP lead to more strong induction of apoptosis. Comparing the treatment with a single drug to the combination treatment, the quantity of cells in the G0 stage increased in case of combination treatment. Caspase-3 activity and mitochondrial membrane potential loss was considerably greater in combined therapy in comparison to treatment of individual drug. The findings of this study support the idea that myricetin and methyl eugenol administered in combination with cisplatin could be a feasible clinical chemotherapeutic strategy for treating human cervical cancer [100].

### 3.11. Lung Cancer

According to a recent study, myricetin was found to inhibit PD-L1 expression in human lung cancer cells that is brought on by IFN. Both the expression of IDO1 and kynurenine production are lowered. Additionally, myricetin restored the survival, proliferation, expression of CD69, and secretion of interleukin-2 in Jurkat-PD-1 T cells that had been suppressed by IFN-exposed lung cancer cells. Myricetin targeted and blocked the JAK-STAT-IRF1 axis, which was the mechanism by which IFN-upregulated PD-L1 and IDO1 at the transcriptional level [101]. The xenografts were created by transplanting A549 cells into immunodeficient mice. In the S4-2-2 (5,7-dimethoxy-3-(4-(methyl(1-(naphthalen-2-ylsulfonyl) piperidin-4-yl amino butoxy-2-(3,4,5-trimethoxyphenyl)-4*H*-chromen-4-one) given group, the tumor weight attenuated and tumor volume inhibition rate was 41.9% than the DMSO group. Additionally, the tumor burden that given S4-2-2 treatment was lower than that in the DMSO control mice. Besides, number of apoptotic cells enhanced after S4-2-2 treatment. Furthermore, invasiveness of A549 cells was prevented and number of migrating cells was decreased after S4-2-2 treatment [102].

After myricetin therapies, A549 and NCI-H446 cells were looked at using a microscope to detect cell swelling and empty cell membranes. Transmission electron microscopy also revealed the existence of multiple pores, another hallmark of pyroptosis, with in cell membranes of myricetin-treated A549 cells and NCI-H446. When considered as a whole, the results show that myricetin causes pyroptosis in NCI-H446 and A549 cells by cleaving GSDME rather than GSDMD [103]. Cytotoxic potential of myricetin in A549 as well as A549-IR cells were examined. A549 IR cells treated with myricetin showed a negligibly cytotoxic activity 48 h after incubation. Additionally, myricetin was found to inhibit the migration of A549-IR cells in a concentration dependent manner. Furthermore, it was observed that A549 IR cells expressed more slug, MMP9, vimentin, and MMP2 while expressing less E-cadherin. Surprisingly, myricetin treatment in A549 IR cells did not significantly increase E-cadherin expression. Slug, MMP2, MMP9, and vimentin expression was all significantly reduced in A549 IR cells after exposure to 100 µM myricetin, in contrast to E-cadherin [104]. Evaluation of the effects of myricetin in combined application with radiotherapy on improving radiosensitivity of lung cancer A549 and H1299 cells. After receiving X-ray exposure in vitro, the myricetin-treated groups showed signs of significantly suppressed cell surviving fraction and proliferation, increased Caspase-3 protein expression, and increased cell apoptosis in comparison to the exposed group without myricetin treatment. The results of the in vivo assay revealed that myricetin treatment of radiation-exposed mice reduced the growth rate of tumour xenografts [105].

### 3.12. Oral Cancer

The oral squamous cell carcinoma SCC-25 and HaCaT cell lines were used to test the anticancer activity of myricetin and naringenin. Myricetin and naringenin both inhibited the growth of SCC-25 cells, but naringenin only targeted cancer cells while leaving HaCaT cells unaffected. Myricetin and naringenin inhibited cell proliferation, but this was not due to the induction of apoptosis; rather, it was due to cell cycle disruption, as G2/M and G0/G1 blockages were noted after 24 h of treatment in HaCaT and SCC-25 cells, respectively. Besides, myricetin induced a reduction of Cyclin B1 in HaCaT and Cyclin D1 in SCC-25 cells. Myricetin and naringenin were both found to be able to decrease the motility of HaCaT and SCC-25 cells in assays for wound healing and cell invasion. The results of the study demonstrate the cancer preventive potential of myricetin.as well as naringenin on oral squamous cell carcinoma as they employ cytostatic effect via the impairment of cell cycle progression. Consequently, naringenin and myricetin seem as hopeful candidate as oral cancer chemo preventive agents [106].

### 3.13. Lymphoma

The anticancer potential of myricetin was investigated based on lymphoma. By directing bruton tyrosine kinase, myricetin was reported to be more sensitive to human diffuse large B lymphoma cell TMD-8 than other tumor cells (BTK). The HTRF assay demonstrated that myricetin inhibited BTK kinase and that it could interact with key residues including Leu408, Thr474, and Ala478 in the BTK active pocket, prevent the autophosphorylation on tyrosine 223, block BTK/AKT signal transduction cascades, and inhibit BTK/ERK. Cell cycle, autophagy, and apoptosis results showed that myricetin could cause the arrest G1/G0 cycle by regulating cyclin B1/D1 expression, initiate autophagy, and cause apoptosis by elevating the ratio of Bax/Bcl-2. Oral myricetin administration significantly inhibited the growth of the TMD-8 xenograft tumour in vivo without causing any negative side effects. Myricetin may also cause tissue lymphoma cells to proliferate less and stimulate apoptosis [107].

### 3.14. Leukemia

Treatment with myricetin exhibits potent pro-apoptotic and anti-proliferative effects on K562 human leukemia cells in a concentration-dependent means. Significantly, exogenous addition of guanosine clearly lowered the cytotoxic effects of myricetin on leukemia cells (K562) cells. Overall, these findings show effective anti-leukemia activity of myricetin because it inhibits the biosynthesis of purine nucleotides by suppressing the catalytic potential of hIMPDH1/2 [108]. Myricetin derivatives such as the 3, 7, 4’, 5’- tetramethyl ether of myricetin (1), isolated from the hexane extract of *Cistus monspeliensis*, and its 3’, 5-diacetyl derivative (2) which was prepared, and the pure compound, myricetin (3), were evaluated for their in vitro cytotoxic potential against human leukemic cell lines. Compound **2** showed greater cytostatic as well as cytotoxic activities as compared to compound **1**, whereas compound **3** was not active against all used cell lines [109].

### 3.15. Bladder Cancer

The least concentration of myricetin was found to have an impact on cell viability in a bladder cancer study. Myricetin treatment negatively affected cell viability in a concentration- and time-dependent manner. In addition, cells subjected to myricetin (40 or 80 µM) showed significantly decreased rates of cell growth. Additionally, myricetin treatments resulted in a concentration arrest of the cell cycle in the G2/M phase for the bladder cancer cell (T24). The control cells displayed intact nuclei, as shown by the microscopic images, whereas the myricetin (40 or 80 µM)-treated cells displayed significant nuclear fragmentation, that is indicative of apoptosis. An in vivo study showed that the tumor growth rate in the myricetin group was lower than that of the control group. According to the findings, myricetin treatment at a dose of 5 mg/kg per day had antitumor effects on bladder cancer xenografts in vivo. Additionally, myricetin treatment demonstrated a greater rate of survival in comparison to the control group [110].

### 3.16. Thyroid Cancer

The proliferation of human anaplastic thyroid cancer (HATCs) cell was reported to be significantly reduced by myricetin nearly 70%. Besides, a significant percentage of dead cells have displayed the arrest of sub-G1 phase. In addition, myricetin demonstrated cytotoxicity and caused condensation of DNA in concentration-dependent ways in human anaplastic thyroid cancer (SNU-80) cells. The stimulation of caspase cascades and the Bax: Bcl-2 ratio at a concentration of 100 µM were both increased involving the myricetin-induced cell death mechanism. Along with altering the mitochondrial membrane potential, myricetin promoted the discharge of an apoptosis-inducing factor into the cytosol from mitochondria [63]. Another study showed that myricetin, in a concentration-dependent manner, caused DNA condensation and cytotoxicity in SNU-790 human papillary thyroid cancer (HPTC) cells. Myricetin also increased the expression ratio of Bax:Bcl-2 and caspase cascade activation. Additionally, myricetin changed the potential of the mitochondrial membrane and started the secretion of the apoptosis-inducing component or factor. These findings support the notion that myricetin causes SNU-790 papillary thyroid cancer cells to die, suggesting that it may be useful in the emergence of therapeutic agents for treating thyroid cancer in humans [111].

### 3.17. Bone Cancer

Osteosarcoma is the most common form of bone cancer. It has been reported that proliferation and DNA replication decreased with myricetin treatment, whereas it increased apoptotic DNA fragmentation in canine osteosarcoma cell lines, DSN and D-17. Moreover, it enhanced generation of ROS, depolarization of MMP and lipid peroxidation, in both used cells. In canine osteosarcoma cells, myricetin intervention stimulated the phosphorylation of p90RSK, p70S6K, AKT, JNK, and ERK1/2. As a result, it was concluded that myricetin could be a highly promising and less harmful treatment approach for the prevention and regulation of canine osteosarcoma progression [112].

### 3.18. Skin Cancer

There are three main types of skin cancer as melanoma, basal cell carcinoma and squamous cell carcinoma. In epidermal JB6 P+ cells of mouse skin, myricetin inhibit the expression of Cox-2 that is brought on by UVB exposure. Besides, the treatment with myricetin prevented NF-κB activation brought on by UVB in a dose-dependent manner as well as activator protein-1 activation. Moreover, myricetin prevented Fyn kinase activity as well as then reduced UVB-induced phosphorylation of MAPK. Myricetin was found to directly inhibit Fyn kinase activity in mouse skin in vivo, which in turn reduced UVB-induced Cox-2 expression. The results of the mouse skin tumorigenesis study clearly showed that pre-treatment with myricetin significantly and concentration-dependently reduced the incidence of UVB-induced skin tumours. Overall, these finding designated that myricetin show powerful chemopreventive potential principally by targeting Fyn in skin carcinogenesis [113]. The anticancer capacity of myricetin has been explored in A431 cell lines of skin cancer. Myricetin has found to have potential anticancer effects against skin A431 cancer cell lines. It had been reported that anticancer properties of myricetin were a consequence of modifications in the membrane potential of mitochondria brought on by ROS and the start of apoptosis. Besides, the response to myricetin treatment resulted in modifications to the Bcl-2 and Bax expressions. In addition to inducing cell cycle arrest in A431 cells, myricetin was further explored to prevent migration and invasion. These findings suggest that myricetin might be an important lead molecule for the discovery of a successful skin cancer therapeutic strategy [114].

### 3.19. Myeloma

Myricetin (10 µM and 20 µM) forms showed negligible degrees of genotoxicity in lymphocytes of multiple myeloma patients in contrast with lymphocytes from healthy individuals, according to an in vitro study. In addition, western blot results showed that lymphocytes from myeloma patients had higher p53 protein levels and lower Bcl-2/Bax ratios than did lymphocytes from healthy people. The regulation of apoptotic proteins activated by myricetin exposure in lymphocytes of myeloma patients occurred via P53 as well as oxidative stress-dependent pathways, as evidenced by the notable increase in intracellular reactive oxygen species level [115].

### 3.20. Brain Cancer

The ability of myricetin to inhibit the proliferation of glioma cells of human (U251) was investigated while also assessing how it affected the production of ROS, the cell cycle, apoptosis, apoptosis-related proteins, and cell migration. Growth inhibitory potential of human glioma cells was observed by myricetin treatment as dose-dependent and time dependent. U251 cells treated with myricetin became detached from surrounding cells, causing clusters of cells to move around in the medium. Besides, apoptotic cell death was initiated by treatment of myricetin. The percentage of early as well as late apoptotic cells increased after treatment with myricetin. Additionally, after myricetin treatment, there was a concentration-dependent reduction in the expression of Bcl-2 and Bcl-xl as well as a raise in Bad and Bax levels. The use of this medication resulted in an arrest of cell cycle in the G2/M phase [116].

In a separate study, myricetin, tumour necrosis factor-related apoptosis-inducing ligand (TRAIL), or both compounds were applied to human astrocytes and glioblastoma cells. Glioma cells quickly underwent apoptosis when treated with subtoxic doses of myricetin in combination with TRAIL. Remarkably, combined treatment consisting of myricetin and TRAIL were not affected on human astrocytes. Combined treatment with myricetin as well as TRAIL improved the effector caspases-3/-7 and activation of initiator caspases-8/-9. Furthermore, over-expression of the short isoforms of bcl-2 and c-FLIP (S) lowered the level of apoptosis induced by the combination of TRAIL and myricetin. In addition, myricetin decreased the degree of expression of both the long and short isoforms of bcl-2 and c-FLIP. Bcl-2 and the short isoform of c-FLIP were reported to be the chief regulators of death of malignant glioma cell associated with TRAIL-myricetin treatment [117].

**Table 2:** Role of myricetin in different types of cancer.

Cancer Type	Dosage	Findings	Refs.
Prostate cancer	0, 25, or 50 µmol/L	Myricetin inhibits migration, invasion, and the EMT in PCa cells.	[60]
	50 μM	Myricetin inhibits the demethylation activity of KDM4A, KDM4B, and KDM4C	[82]
Colon cancer	100 μM	In comparison to controls, treatment with 100 μM of myricetin induced about 70% reduction in cell viability. Furthermore, treatment of HCT-15 with 100 μM of myricetin induced significant nuclear rounding and shrinkage of HCT-15 human colon cancer cells in comparison to controls	[85]
	0–200 μM	The results demonstrate that HCT-15 cell viability rate to be 100%, 36.8%, 35%, and 31.4% in cells treated with 0, 100, 150, and 200 μM myricetin, respectively. Whereas, the viability rates of CCD-18co cells showed 100%, 78.9%, 76.1%, and 49.9% at 0, 100, 150, and 200 μM myricetin, respectively	[86]
	50 and 100 μmol/L	Light microscopy-based results showed that the apoptotic cells became rounder and smaller. Moreover, there were vesicles in the cell membrane and apoptotic bodies in the cell. The percentage of apoptotic cells was 28.5 and 67.4%.	[57]
Liver cancer	0, 100 or 200 μM	Myricetin treatment significantly decreased cell growth and induced visible cell death in HCC cells. Treatment of HepG2 and Huh-7 cells markedly inhibited cell growth. Furthermore, myricetin-treated HepG2 cells showed increased apoptosis rate compared to control cells. Similar effects of myricetin on apoptosis were detected in Huh-7 cells.	[88]
	100 µM	The cell scratch assay indicated that compared with the control group, the migration of MHCC97H cells was inhibited when the cells were treated for 24 and 48 h. Furthermore, real time-quantitative polymerase chain reaction (RT-qPCR) analysis showed that the relative mRNA expression of E-cadherin in MHCC97H cells significantly enhanced at 25 µM myricetin and the relative mRNA expression level of N-cadherin significantly reduced at 100 µM, along with that of vimentin	[89]
	0–50 μM	HCC cells treated with myricetin were incubated with the CCK-8 reagent. The results showed that the viability of these cancer cells treated with this compound was obviously declined in a dose-dependent manner	[61]
Gastric cancer	0–30 μM	The cell viability of gastric cancer cells was determined after 24 h of treatment with 0–30 μM myricetin. The cell viability of these cancer cells was 95.8% with 5 μM myricetin, 90.3% with 10 μM myricetin, 80.6% with 15 μM myricetin, 64.6% with 20 μM myricetin, 52.7% with 25 μM myricetin, and 36.3% with 30 μM myricetin. Furthermore, apoptotic bodies in these cancer cells at dose of of 0, 15, and 25 μM for 24 h, showed a significant increase in the number of apoptotic bodies.	[58]
	20 and 40 mol/L	The effect of myricetin on apoptosis in HGC-27 and SGC7901 cells was determined. It was observed that the percentage of apoptotic cells was higher. As the concentration of myricetin increased, the percentage of apoptotic cells increased. Furthermore, the anti-apoptotic protein Bcl-2 and pro-caspase-3 were significantly decreased and increased concentration of myricetin increased the degree of low expression.	[64]
Pancreatic cancer	0, 12.5–200 µM	It significantly reduced cell viability in all pancreatic cancer cells tested. In addition, incubation of MIA PaCa-2 and S2-013 in culture medium containing myricetin for 6 h resulted in a statistically significant increase in caspase-3 and 9 activities.	[90]
Bile duct cancer	5, 10 and 25 μM	The treatment of myricetin for 24 h inhibited migration and invasion of KKU-100 cells by restraining the phosphorylation of STAT3 and its downstream factors including ICAM-1 and MMP-9 as well as some inflammatory-associated genes such as iNOS and COX-2	[49]
Esophageal cancer	0–100 µM	Treatment of the EC9706 cells with different concentration 5-FU alone could lead to inhibition effect on clonogenic survival. However, when combined with different concentration of myricetin, the surviving fraction decreased significantly	[66]
	20 or 40 μM	It resulted in the inhibition of the proliferation of EC9706 and KYSE30 cells in a dose- and time dependent manner. EC9706 and KYSE30 cells were treated with myricetin and DMSO and were placed in a transwell chamber. The fraction of KYSE30 cells in the G0/G1 phase of the cell cycle in the 20 μM myricetin, 40 μM myricetin, and DMSO groups were 65.41, 70.93, and 56.38%, respectively	[67]
Ovarian cancer	10, 20 and 40 μM	ROS levels in SKOV3 cells were significantly reduced by myricetin in a dose dependent manner. Relative to controls, intracellular MDA was significantly reduced dose-dependently by myricetin, while SOD levels increased significantly. LDH levels in the culture medium also reduced significantly	[69]
	25 µM	Induction of apoptosis in A2780 and OVCAR3 cells was observed. In A2780 cells treated with myricetin, an ~2.5-fold increase in the apoptotic signal was observed, whereas, the apoptotic signal was increased by ~4-fold in OVCAR3 cells.	[59]
	40 µg/mL	The nuclei of myricetin-treated cells appeared more condensed, when compared with the untreated cells	[95]
Breast cancer	54 μM	The results showed a significant increase in the apoptosis rate in MCF-7 cells treated with myricetin.	[96]
	0, 5, 10, 15, 20, and 25 µM	The SK-BR-3 cells treated with different doses of myricetin exhibited viabilities of 96.5% at 5 µM, 78.1% at 10 µM, 51.4% at 15 µM, 42.5% at 20 µM, and 37.9% at 25 µM. Thus, the SK-BR-3 cells showed a dose-dependent decrease in viability compared to that of the control group	[99]
Cervix cancer	10–100 µM	Cell viability with different treatments in addition to myricetin showed decreased cell viability	[100]
Lung cancer	30 µM	Myricetin significantly inhibited cell migration and invasion of cells from the upper chamber to the lower chamber, and inhibited the invasion of cells were significantly higher than that in the control group.	[103]
Oral cancer	50–250 mM	SCC-25 proliferation was inhibited, in fact, for doses above 150 mM decrease of cell viability to values below 40%. After 72 h, the growth inhibitory effect persisted for the highest concentration of myricetin (250 and 200 mM), whereas for the intermediate dose, myricetin 150 mM, a partial restoration of cell viability was observed	[106]
Lymphoma	5, 10, 20, and 40 μM	Myricetin arrest the cell cycle in the G1/G0 phase in a dose-dependent manner, and the proportion of cells in the S phase was decreased. Precisely, treatment with 40 μM myricetin meaningfully increased the proportion of cells in the G1/G0 phase from 43.00 ± 1.25% to 62.67 ± 4.95%, and the proportion of cells in the S phase decreased from 37.10 ± 0.52% to 25.30 ± 1.05%	[107]
Leukemia	20 and 50 µM	It displayed an arrest of cells in the S-phase in a dose-dependent manner.	[108]
Bladder cancer	20–100 μM	Reduction in cell viability with myricetin treatment at concentrations of after 12 h ranged from 2.6% to 61%, whereas after 24 h and 48 h ranged from 2.9% to 70% and 3% to 80%, respectively. However, treatments with myricetin (0–80 µM) for 24 h resulted in a dosage-dependent arrest of T24 cells in G2/M phase of cell cycle.	[110]
Thyroid cancer	0–100 μM	It reduced cell viability of SNU-80 HATC cells in a dose-dependent manner. In comparison to the control cell treated with 100 μM of myricetin induced about 85% reduction in cell viability on SNU-80 HATC cells	[63]
	25 to 100 mM	It was cytotoxic to SNU-790 HPTC cells in a dose-dependent manner at concen- trations range. In comparison with control cells treated with 100mM myricetin exhibited an approximately 82% reduction in viability.	[111]
Bone cancer	0–100 μM	It gradually decreased the cell proliferation of D-17 and DSN cells in a dose dependent manner, as proliferation of D-17 and DSN cells reduced to 40.3% and 38.2%, respectively, after treatment with 100 μM myricetin, as compared to vehicle-treated control cells	[112]
Skin cancer	2.5–20 μM	It inhibited UVB-induced COX-2 protein expression and promoter activity in a dose-dependent manner	[113]
	0–40 μM	It caused apoptotic cell death in A431cells dose-dependently. The apoptotic A431 cells augmented from 1.25% in control to 46.3% at 40 μM of myricetin.	[114]
Myeloma	10–20 µM	Comet assay demonstrated that myricetin bulk (10 µM) and nano (20 µM) forms exhibited a non-significant level of genotoxicity in lymphocytes from multiple myeloma patients when compared to those from healthy individuals.	[115]
Brain cancer	0, 15, 60 and 120 μM	increasing doses of myricetin, led to dose-dependent increase in Bax and Bad levels and a dose-dependent decrease in Bcl-2 and Bcl-xl expression levels	[116]
	0–150 μM	Combining TRAIL with Myricetin led to a significant increase in cleaved fragment of PARP and active cleaved caspase-3/-7/-8/-9 in both U251 and NCH89	[117]

## 4. Pharmacokinetic and Strategies to Improve Efficacies of Myricetin

Several studies reconnoiter the pharmacokinetic properties of myricetin using different method. Myricetin has been identified and quantified in rat plasma after oral and intravenous administration using a precise and sensitive ultra-performance liquid chromatography-tandem mass spectrometry (UPLC-MS/MS) method. For selectivity, precision, linearity, accuracy, recovery, stability, and matrix effect, the developed method received approval. Over a broad concentration range of 2–4000 ng/mL, the assay was validated. Precisions within (intra) and between (inter) days all were under 13.49%, and the range of accuracy was from 95.75 to 109.80%. In order to analyze a pharmacokinetic investigation of myricetin after intravenous and oral administrations to rats, the current methodology was successfully used. Myricetin was found to have an absolute bioavailability of 9.62% and 9.74% at 2 oral concentrations, indicating that it was poorly absorbed after oral route administration [118]. Additionally, factors such as its low water solubility, decreased stability in gastrointestinal fluid, and rapid in vivo biotransformation could all contribute to its low absorption [119]. The role of myricetin on the pharmacokinetics of losartan and its active metabolite, EXP-3174, were examined in rats. After oral administration of losartan (0.4, 2 and 8 mg/kg) to rats in the condition of having or not having of myricetin, the pharmacokinetic parameters of both losartan and EXP-3174 were assessed. It was evaluated how myricetin affected the activity of CYP2C9, CYP3A4, and P-glycoprotein. CYP2C9 and CYP3A4 enzyme potential were inhibited by myricetin at concentrations of 50% inhibition of 13.5 and 7.8 μM, respectively. In addition, myricetin significantly and concentration-dependently increased the cellular accumulation of rhodamine 123 in MCF-7/ADR cells overexpressing P-glycoprotein. Myricetin have been found to significantly alter the pharmacokinetic parameters of losartan in contrast to the control group. Losartan’s peak plasma content increased by 31.8–50.2% and the area under the plasma concentration-time curve by 31.4–61.1%, respectively, when myricetin (2 or 8 mg/kg) was present. As a result, in comparison to the control, myricetin significantly increased the absolute bioavailability of losartan. Further evidence that myricetin could inhibit the CYP-mediated metabolism of losartan to its active metabolite was provided by the 20% reduction in the metabolite-parent area under the plasma concentration-time curve ratio that was caused by concurrent use of the myricetin drug [120].

Its pharmacological potential in several diseases have been confirmed including cancer through modulation of cell signalling molecules activities. Irrespective of its potential benefits, its role in diseases management is limited due to low bioavailability and low absorption, quick elimination. Accordingly, a number of discoveries were demonstrated to get around the problems caused by poor bioavailability, low absorption, and quick elimination. Different types of nano-formulation and its role in the improvement of its efficacies are described (Table 3). Nano based formulation has been prepared and their role in cancer inhibition tested and result confirms that these formulations can be employed as delivery to improve the bioavailability of this compound. Polymeric carrier based on chitosan-functionalized Pluronic P123/F68 micelles loaded with myricetin was prepared to expand the therapeutic index of chemotherapy of glioblastoma cancer. The outcomes confirmed that myricetin-loaded micelles (MYR-MCs) displayed enhanced cellular uptake as well as antitumor potential as compared to free myricetin in vitro, with a meaningfully improved anticancer potential in vivo subsequent effective transport across the blood-brain barrier. However, myricetin-loaded micelles did not disturb the barrier function, brain endothelial, the liver, kidneys or heart. In addition, myricetin-loaded micelles altered the expression of apoptotic proteins in mice. In conclusion, myricetin-loaded micelles may be measured an active as well as hopeful drug delivery system in the glioblastoma treatment [121]. A combination of multidrug resistance protein (MRP-1) siRNA and myricetin (Myr)-loaded mesoporous silica nanoparticles (MSN) was created. In comparison to non-targeted nanoparticles, folic acid-conjugated nano-formulations showed a significant uptake in lung cancer cells. In vitro research on drug release suggested that sustained release in folic acid-conjugated mesoporous silica nanoparticles (MSN) with myricetin and MRP-1 nanoparticles was preferable to free myricetin and MSN combined with multidrug resistance protein (MRP-1)/Myr. The viability of the lung cancer cell lines was noticeably decreased by treatments using FA-conjugated MSN in combination with myricetin and MRP-1, which was accompanied by a decreased rate of colony formation. In addition, myricetin and MRP-1-loaded mesoporous silica nanoparticles significantly induced apoptosis in cells of lung cancer. The ability of FA-conjugated mesoporous silica nanoparticles with myricetin and MRP-1 nanoparticles to accurately gather at tumour sites was demonstrated by in vivo fluorescence research. FA-conjugated MSN equipped with myricetin and MRP-1 nanoparticles have been suggested to more effectively inhibit tumour growth with negligible adverse consequences when compared to standard myricetin and mesoporous silica nanoparticles coupled to MRP-1/Myr nanoparticles [122]. Myricetin-loaded NLCs was reported to reduce cell viability by 50 ± 2.3 to 40 ± 1.3%. Myricetin-loaded NLCs and docetaxel administered together resulted in a higher percentage of breast cancer cells undergoing apoptosis (MDA-MBA231). Proapoptotic factor Bax and Bid mRNA rates increased in response to expression of antiapoptotic genes, while proapoptotic factor Bax and Bid rates markedly decreased. Finally, evidence suggests that the NLC delivery mechanism may be a promising strategy for enhancing the impact of anticancer drugs like docetaxel on breast cancer [123].

Microparticles containing myricetin solid lipid nanoparticles (SLNs) were prepared for lung cancer therapy. Greater antitumor potential of myricetin-phospholipid-complex as well as 3-fold decrease in IC_50_ were accomplished with myricetin- solid lipid nanoparticles. This might be related to increased fluorescence intensity and greater cellular uptake as seen by confocal imaging. Spray-drying was used to create respirable microparticles from solid lipid nanoparticles encasing MYR-PH-CPX. In addition to good flowability and >80% release over 8 h, the latter validated MMAD of 2.39 mm and a span index of 1.84. The study emphasizes the phospholipid-ability complexes to encapsulate myricetin at the nanoscale, its antitumor properties, and its cellular uptake. Possibilities for efficient lung carcinoma treatment are provided by the formulation of respirable microparticles [124].

Another recent study was performed to prepare a simple as well as stable synthesis of gold nanoparticles (AuNPs) with myricetin via ultrasound-assisted method. Result revealed that anticancer potential by myricetin- gold nanoparticles -treated cells displayed a good proportion of dead cells demonstrated with formation of pro-apoptotic bodies. Furthermore, myricetin-AuNPs showed depolarization of mitochondrial membrane potential as well as production of ROS. This study evidences that myricetin-gold nanoparticles (NPs) hold great potential to use against breast cancer as a strong anticancer drug [125]. To encapsulate myricetin, folic acid (FA)-conjugated bovine serum albumin (BSA) NPs were used. It was investigated how to deliver myricetin to breast cancer cells that were folate receptor-positive using naturally overexpressed folate receptor. The viability of the MCF-7 cells was effectively decreased by FA-myricetin-BSA NPs. Additionally, this formulation increased myricetin uptake in MCF-7 cells. Following incubation, it was possible to see the typical distorted membrane bodies and condensed nuclei associated with apoptosis. The observed outcomes demonstrate that the newly created FA-myricetin-BSA NPs may serve as a potential myricetin carrier to enhance the anticancer potential of this anticancer drugs [126].

## 5. Synergistic Effects of Myricetin with Anti-Cancer Drugs

**Figure 4:**
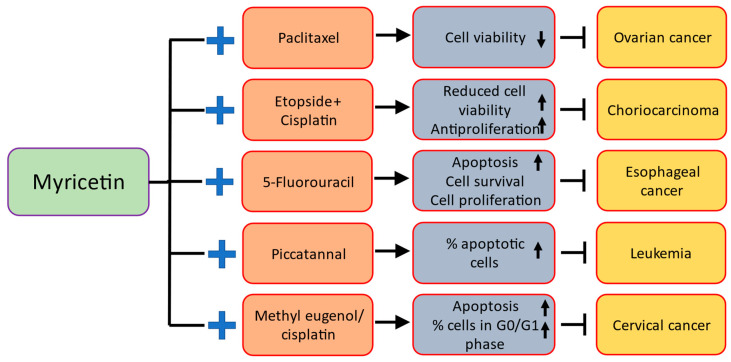
Synergistic effects of myricetin with anti-cancer drugs.

Anti-cancer drugs show a significant role in cancer treatment but their potential role is restricted due to their adverse effects. Natural compounds either native or its bioactive compound enhance the activity of anti-cancer drugs based on various cancer cells via synergistic antitumor effect with less or no toxic effects. To determine if myricetin can increase the ability of paclitaxel to act as a chemotherapeutic agent. In direction to examine whether myricetin is capable to improve the chemotherapeutic potential of paclitaxel. Synergistic effects of myricetin with anti-cancer drugs are described below (Table 4 and Figure 4, refs. [59,70,92,100,129]).

In order to investigate whether myricetin is able to enhance the chemotherapeutic potential of paclitaxel (PTX), the ovarian cancer cells were treated with a sub-lethal concentration of myricetin (5 µM) for 48 h, and then incubated with a sub-lethal concentration of paclitaxel (100 nM). The findings showed that paclitaxel (100 nM) did not significantly promote cytotoxicity in the cell types used. However, a discernible decline in cell viability was observed when the myricetin-treated cells were further incubated with paclitaxel (100 nM). Thus, the combined treatment of paclitaxel and myricetin was effective in used cell lines, resulting in a loss of cell viability of approximately 50% [59]. It was investigated whether myricetin could improve the radiosensitivity of lung cancer H1299 and A549 cells when used in combination with radiotherapy. Relying on in vitro study, the groups administered with myricetin showed significantly reduced cell surviving percentage as well as improved cell apoptosis, proliferation, and elevated Caspase-3 protein expression in comparison to the exposed population not having myricetin therapies.

In vivo research also showed that myricetin treatment of radiation-exposed mice significantly slowed the growth of tumour xenografts [105]. Myricetin or methyl eugenol combined with cisplatin had a positive influence than either drug alone at inhibiting cancer cell growth and inducing apoptosis. Further, this combination was found to significantly increase the induction of apoptosis. In addition, when compared to single drug therapy, this combination therapy substantially boosted the number of cells in the G0/G1 stage. In addition, the combined treatment significantly increased the activity of caspase-3 and mitochondrial membrane potential loss. The results of this study point to myricetin and methyl eugenol as a potential clinical chemotherapy strategy for treating human cervical cancer [100].

In (HL-60) leukemia cells, myricetin or piceatannol alone caused apoptotic cell death in a way that depended on the concentration and the duration of exposure. Likewise, the percentage of apoptotic (HL-60) leukemia cells was noticeably higher in the combined study population. After piceatannol therapies, the proportion of TUNEL-positive HepG2 cells was significant, but it was even lower in the combined treatment than in the piceatannol-only-treated cells. In summary, (HL-60) leukemia cells-initiated apoptosis in response to piceatannol and myricetin, but HepG2 cells did not (hepatoma) [129]. Choriocarcinoma cells-based study reported that myricetin showed synergistic antiproliferative potential with chemotherapeutics, etoposide and cisplatin [70]. To determine whether myricetin has an encouraging inhibitory impact when used together with 5-FU, a study was conducted using esophageal cancer cells. When compared with the 5-FU group without treatment of myricetin, 5-FU treated groups with combine with myricetin displayed meaningfully suppressed cell survival fraction as well as proliferation, apoptosis of cells was increased. The 5-FU combined with myricetin groups showed a lower cyclin D, survivin, Bcl-2, and increased caspase-3, p53 expression levels. Furthermore, in an in vivo study, mice given the mixture of 5-FU and myricetin showed a significant reduction in the rate of tumour xenograft growth [92].

## 6. Toxicity of Myricetin

Numerous reports have proven that myricetin shows numerous pharmacological activities including cancer. Myricetin has recognized generally safe. Intraperitoneal-injection of myricetin at an extreme dose of 1000 mg/kg did not cause death of mice, easing the safety issue [131] and this flavonols at approximately 0.5 LD50 doses suppressed the vascular endothelial growth factor (VEGF)-stimulated HUVEC tubular structure formation [132]. Few reports have reported toxic side effects under specific conditions. Isolated guinea pig enterocytes were exposed to kaempferol, quercetin, and myricetin in concentrations of 50–450 μM. Toxicity was evaluated using trypan blue exclusion and lactic dehydrogenase (LDH) leakage. Myricetin caused cellular damage at 450 μM as compared with a control incubation; cellular viability was 12–60% lower and LDH leakage 28–41% greater after 3 h of incubation. Furthermore, quercetin and myricetin, both of which produce superoxide on autoxidation, seemed to be more toxic than kaempferol [133]. Myricetin is susceptible to autooxidation at pH over than 7.4 which could cause to the release of reactive oxygen species and can cause a toxic effect on the biomolecules [134].

## 7. Limitations and Future Prospects

Myricetin possess significant health benefits and despite its recognized role in cancer management, scanty data is available to support its thorough clinical implications [135]. Some major limitations of this compound in the treatment of diseases including cancer are due to its low water solubility, fast metabolism and quick excretion from the body. In addition, its role in cancer treatment and prevention is still to be explored thoroughly due to its low bioavailability. Besides this, the therapeutic challenges of myricetin are its lack of translational studies to know the exact route of administration, and effective dosage for different cancers.

In recent times, the clinical investigations have started to fulfil the gap between the research laboratory experiments and their direct clinical implication. The researchers are now exploring the clinical uses of flavonoids as anticancer agents in different clinical trials. Furthermore, nano-formulation based flavonoids are gaining interest to target different tumors to minimize the off-target side effects and enhancing the drug efficacy. However, all these investigations are under pre-clinical stage [136].

The future prospects of the usage of this compound includes more research to be done based on different clinical trials to understand the action mechanism, safety, and proper dosage. Moreover, different challenges should be focused on engineering different nano-formulations of myricetin to overcome the poor bioavailability, loading capacity, targeted orientation and premature release of this compound. Furthermore, some derivatives of myricetin need to be synthesized to check their anticancer potential.

## 8. Conclusions

Cancer is one of the main culprits of death worldwide. In spite of the progress of treatment approaches, cancer leftovers a crucial cause of death globally. Moreover, current mode of treatment of cancer is expensive and causes adverse effects. Medicinal plants or bioactive compounds are rich source of antioxidant and such property shows role in diseases cure and prevention. The concentration of myricetin content varies significantly (10–1600 mg/kg) between different plants and vegetables. Myricetin is a flavonoid and its role in health management have been confirmed as hepatoprotective, anti-inflammatory, neuroprotective and cardioprotective. Moreover, its role in cancer prevention has been proven as it suppresses the cancer growth, inhibit angiogenesis, regulate cell cycle, inhibit inflammation, induces apoptosis and modulates various other cell-signalling molecules. The myricetin used in different cancer cell lines significantly varies (5–200 μM) to evaluate its anticancer potential. Nano based formulation has been prepared and their role in cancer inhibition tested and result confirms that these formulations can be employed as delivery to improve the bioavailability of this compound. However, different challenges should be focused on engineering different nanoformulations of myricetin to overcome its poor bio-availability, loading capacity, targeted orientation and premature release of this compound. Furthermore, some derivatives of myricetin need to be synthesized to check their anticancer potential. Besides, synergistic effects with anti-cancer drugs have been proven through increased the induction of apoptosis and reduced the cell viability. To identify the precise effectiveness of this novel compound in preventing and treating cancer, extensive future studies based on in vivo or clinical studies are indeed recommended.

## Figures and Tables

**Table 3:** Nano-formulation and its role in cancers.

Nanoformulation/ Derivatives	Cancer	Findings	Refs.
Myricetin-loaded micelles	Glioblastoma	Myricetin-loaded micelles showed role in the enhancement of cellular uptake and antitumor potential in comparison of free myricetin	[121]
FA-conjugated MSN combined with myricetin as well as MRP-1	Lung cancer	This formulation significantly reduced the cell viability, and caused induction of apoptosis in lung cancer cells	[122]
Myricetin-loaded NLCs	Breast cancer	NLC delivery vehicle may be an encouraging strategy to enhance the efficacy of docetaxel on breast cancer.	[123]
Myricetin-solid lipid nanoparticles	Lung cancer	Enhanced antitumor potential and 3-fold in IC_50_	[124]
Myricetin-gold nanoparticles	Breast cancer	Myricetin- gold nanoparticles showed great potential against breast cancer	[125]
FA-Myr-BSA NPs	Breast cancer	This nanoformulation efficiently reduced the cancer cells viability.	[126]
S4-10	Lung cancer	Myricetin derivatives, has displayed the highest antitumor efficacy in dose-dependent manner. The proliferation of A549 cells were significantly attenuated by S4-10 in both in vitro and in vivo assays.	[127]
S4-2-2	Lung cancer	Different myricetin were synthesized and tested. Experiments on non-small cell lung cancer (NSCLC) showed that S4-2-2 (5,7-dimethoxy-3-(4-(methyl(1-(naphthalen-2-ylsulfonyl) piperidin-4-yl) amino) butoxy)-2-(3,4,5-trimethoxyphenyl)-4*H*-chromen-4-one) had the strongest effect on A549 cell inhibition across all compounds.	[102]
3, 7, 4′, 5′-tetramethyl ether (1) and 3′, 5-diacetyl derivative (2)	Leukemia	Two myricetin derivatives, were examined for their in vitro cytotoxic activity against human leukemic cell lines. Compound **2** exhibited higher cytostatic and cytotoxic activities in comparison to compound **1** with vinblastine used as a control.	[109]
M10, a novel derivative of Myricetin	Colorectal cancer	Oral administration of this derivative M10 exerts chemoprevention of ulcerative colitis and colorectal tumor in mice	[128]

**Table 4:** Synergistic effects of myricetin with anti-cancer drugs.

Cancer	Cell Lines	Anti-Cancer Drugs/Treatment Type	Outcome of the Study	Refs.
Ovarian cancer	A2780 and OVCAR3	Paclitaxel	When 100 nM paclitaxel was added to myricetin-exposed cells, there was a significant decline in cell viability.	[59]
Lung cancer	A549 cells and H1299	Radiotherapy	Myricetin and radiotherapy together can make pulmonary carcinoma tumors more radiosensitive.	[105]
Cervix cancer	HeLa	Cisplatin	Myricetin, cisplatin, and methyl eugenol, when used in combination, significantly increased the percentage of cells in the G0/G1 phase compared to when used alone.	[100]
Leukemia and hepatoma	HL-60 and HepG2	Piceatannol	The proportion of apoptotic HL-60 (leukaemia) cells was substantially greater in the combination therapy. Only after piceatannol treatment did the percentage of TUNEL-positive HepG2 cells rise significantly; while combined treatment was used, it was even lower compared to piceatannol-only-treated cells.	[129]
Choriocarcinoma	JAR and JEG-3	Etoposide and cisplatin	Myricetin showed synergistic antiproliferative effects with chemotherapeutics, etoposide as well as cisplatin	[70]
Esophageal carcinoma	EC9706	5-fluorouracil	The groups given myricetin and 5-fluorouracil together showed significantly reduced cell survival percentage and proliferation, as well as increased apoptosis.	[92]
Prostate cancer	C4-2B	Enzalutamide	The combination of PLGA-encapsulated myricetin with enzalutamide is potentially effective for castration-resistant prostate cancer	[82]
Cervical cancer	HeLa	Methyl eugenol	Combination of myricetin or methyl eugenol with cisplatin resulted in more potent apoptosis induction.The combination treatment also increased the number of cells in G0/G1 phase dramatically as compared to single drug treatment.	[100]
Lung cancer	A549	cucurbitacin E	Cucurbitacin E-myricetin (CuE: 0.5 µM, myricetin: 20 µM), a combination of these compounds inhibited lung cancer cell proliferation and colony formation, and induced apoptosis and cell cycle arrest in the G0/G1 phase, exhibiting a synergistic effect.	[130]

## Data Availability

Not applicable.
